# Institutional Organisation in America

**Published:** 1914-08-01

**Authors:** 


### INSTITUTIONAL ORGANISATION IN AMERICA.*

### III. Reforms in Gynæcological Out-Patient Clinics.

In one of the previous articles of this series the efforts of the New York Associated Out-patient Clinics to bring about greater uniformity and a higher standard in clinic methods have been illustrated in connection with clinics for children. Still more recent recommendations have now been issued for the model administration and equipment of gynæcological departments, drawn up by the executive committee of the gynæcological section of the New York Association.† The instructions are as full of interest and value as those in respect of institutions for children, as will be seen from the following list of them:

### Duration of Staff Appointments.

1. "The services of physicians in gynæcological clinics shall be limited to four years; but this shall not preclude reappointments." The system of making all medical staff appointments for five-year periods has been adopted here and there in this country, and has before now been advocated in The Hospital. The movement cannot be said to have made much headway yet, but in our opinion it is purely a matter of time before it supersedes the now prevailing practice. Four years is, in our view, too short a term, except possibly for a first appointment; five, or even seven, years is a preferable period.

2. "The value is emphasised of accurate clinical histories, and it is recommended that uniform forms of histories be adopted by all the gynæcological departments of the city." This recommendation, sound enough in itself, is one of comparatively minor importance. There would probably be no great difficulty in getting it adopted, since it is observed in the spirit, if not in the letter, pretty generally already.

3. "In order to equalise the duties and responsibilities of the assistants in the clinics with reference to the taking of histories a weekly rotation system should be adopted, and efforts should be made to secure the services of medical student clerks or other clerical assistants for that purpose."

### Dispensary Equipment.

4. "Local gynæcological treatment is of undoubted value, and each dispensary or out-patient department of a hospital should have a gynæcological department." This and the preceding recommendation call for no special comment. They seem to point to the existence in New York of clinics or institutions where the standard of gynæcological work is not high.

5. "Every properly equipped dispensary should have, in addition to the usual gynæcological instruments, an equipment for cystoscopy; also arrangements for the proper examination of (*a*) urine, (*b*) smears, (*c*) Wassermann reaction, (*d*) blood, (*e*) specimens of tissue for diagnosis, in properly equipped laboratories." With this recommendation it is impossible to disagree; but it must be confessed that, apart from the gynæcological departments of the big general hospitals to which schools are attached, few English clinics reach the standard here indicated. Want of funds is the reason for these deficiencies, no doubt, but a contributory cause is want of courage on the managing boards and want of initiative among the medical staffs.

6. "Gonorrhœa in women should be treated in the department of gynæcology, but syphilitic cases should be referred to the department of dermatology or to a special department of syphilography if one exists." In the present state of scientific knowledge it is certainly desirable that the first of these two above recommendations should be accepted.

### The Province of the Dermatologist.

Gonorrhœa in women is such a local disease, in ninety-nine cases out of a hundred, and its complications result so uniformly in pelvic diseases which are indisputably the province of gynæcologists, that it is quite proper that its treatment should be handed over to them. In case of remote sequelæ or complications arising (ophthalmic, for instance), the assistance of the department concerned could, of course, be secured. With respect to the second part of the paragraph, it is equally desirable that when a special department for syphilis exists, cases should be handed over to it; but we have never quite sympathised with the claim of the dermatologist to be entrusted with the care of this disease. Syphilis is a general blood infection, in which skin eruptions are frequent but not invariable: the great majority of the symptoms do not involve the skin, and if any department has an indefeasible claim to appropriate this disease it is, in our opinion, that of medicine. In this country the surgeons have been until recently inclined to regard syphilis as their exclusive domain; logically we are not in the least disposed to admit the claim.

7. "Children suffering from vaginitis should be treated in the gynæcological department of dispensaries, or in special departments for the treatment of the disease, if such are available." This, again, is a recommendation with which no one is likely to quarrel.

8. "The executive committee wishes to emphasise the value and need of printed hygienic instructions to patients suffering from gonorrhœa and cancer, and recommends that uniform printed instructions be adopted by all gynæcological clinics of the city. The committee also recommends that instruction with reference to cancer of the womb be given to every patient in gynæcological clinics, and that the dispensaries located in districts with a con-

* Articles I. and II. appeared on July 11 and 18 respectively.

† *Medical Review of Reviews*, May 1914, p. 242.

siderable foreign population print all their hygienic instructions in such foreign languages as are most suitable for their neighbourhood." Something, though not enough, has been done in this country along the lines suggested in this recommendation. It has to be recognised that the routine distribution of printed leaflets has disadvantages, such as the unwillingness of out-patients to read them, and their inability to understand and profit by them, even if they do read them. Moreover, a leaflet may tell one patient too much and another too little; throw one into a groundless panic, and quite fail to impress another with whom the spoken word might carry more weight.

Limiting Numbers and Evening Clinics.

9. "The principle of the limitation of the number of patients in each clinic should be adopted, such limitation to be based upon the estimated facilities in men and equipment of each clinic. It is further advised that, as a general working rule, the average of six patients, old and new, per table per hour be adopted." We have already discussed and approved this principle in dealing with the question of children's out-patient clinics in a previous article of this series (The Hospital, July 11, p. 411).

10. "Evening clinics for patients working during the day should be adopted." To the best of our knowledge there is no London hospital which runs an evening clinic for the benefit of employed women; but the experiment of starting one in an industrial neighbourhood would be well worth while, and we should very much like to see it tried.

There is no body in Britain, medical or lay, which corresponds to the New York Associated Out-patient Clinics, whose reports have been analysed above and in previous articles. This is not due, it is hardly necessary to say, to any freedom from defects of British institutions; but rather to a lack of that Yankee energy and determination to improve at which the rest of the world is so often amazed. It is our deliberate opinion that there is room for some such organisation in England, and particularly in London; and that the institutions themselves, the sick poor, and the medical profession would derive great advantages from it. It rests with the more energetic and enlightened of our hospital chairmen, and with the more progressive spirits on the medical staffs, to bring into being an association of this sort and to make it a living force. Self-criticism, self-help, self-improvement are infinitely more palatable processes than interference from politicians with axes to grind. The former would be secured by an imitation of the New York plan; the latter may come about in time if the lesson is not taken to heart.

